# Antimicrobial Resistance Trends among Community-Acquired Respiratory Tract Pathogens in Greece, 2009–2012

**DOI:** 10.1155/2014/941564

**Published:** 2014-01-27

**Authors:** Sofia Maraki, Ioannis S. Papadakis

**Affiliations:** Department of Clinical Microbiology, Parasitology, Zoonoses and Geographical Medicine, University Hospital of Heraklion, 711 10 Heraklion, Crete, Greece

## Abstract

The aim of the present study was to determine the antimicrobial resistance trends of respiratory tract pathogens isolated from patients with community-acquired respiratory tract infections (CARTIs) in Crete, Greece, over a 4-year period (2009–2012). A total of 588 community-acquired respiratory pathogens were isolated during the study period. *Streptococcus pneumoniae* was the most common organism responsible for 44.4% of CARTIs, followed by *Haemophilus influenzae* (44.2%) and *Moraxella catarrhalis* (11.4%). Among *S. pneumoniae*, the prevalence of isolates with intermediate- and high-level resistance to penicillin was 27.2% and 12.3%, respectively. Macrolide resistance slightly decreased from 29.4% over the period 2009-2010 to 28.8% over the period 2011-2012. Multiresistance was observed among 56 (54.4%) penicillin nonsusceptible isolates. A nonsignificant increase in resistance of *H. influenzae* isolates was noted for **β**-lactams, cotrimoxazole, and tetracycline. Among the 67 *M. catarrhalis* tested, 32 produced beta-lactamase and were resistant to ampicillin. Macrolide resistance decreased over the study period. All isolates were susceptible to amoxicillin + clavulanic acid, chloramphenicol, rifampicin, and the fluoroquinolones. Although a decreasing trend in the prevalence of resistance of the three most common pathogens involved in CARTIs was noted, continuous surveillance of antimicrobial susceptibility at the local and national level remains important, in order to guide appropriate empirical antimicrobial therapy.

## 1. Introduction

Community-acquired respiratory tract infections (CARTIs) such as acute otitis media, acute sinusitis, acute exacerbations of chronic bronchitis, and community-acquired pneumonia are very common causes of morbidity and are among the leading reasons for physicians' office visits worldwide [1]. Due to the severity of these infections and the difficulties in determining the bacterial etiology and the antimicrobial susceptibility, treatment is often empirical, consisting usually of orally administered agents. The choice of empiric antimicrobial chemotherapy is guided by the clinical presentation, the severity of the infection, and epidemiologic data, comprising the causative organisms and their susceptibility to antimicrobial agents [2, 3].

*Streptococcus pneumoniae*, *Haemophilus influenzae,* and *Moraxella catarrhalis* are the major pathogens implicated in community-acquired respiratory tract infections. The increasing prevalence of antimicrobial resistance among these species remains a global problem complicating the management of CARTIs [4, 5].

Of particular concern are the emergence and dissemination of *S. pneumoniae* strains resistant to penicillin and multidrug resistant (MDR) to several antibiotic classes [6]. In *H. influenzae* and *M. catarrhalis* *β*-lactamase (BL) production has been the primary mechanism for bacterial resistance to beta-lactams [7, 8]. However, rare *β*-lactamase negative ampicillin-resistant (BLNAR) *H. influenzae* isolates have been reported [7].

In the present study, we describe the antimicrobial susceptibility patterns of community-acquired respiratory tract pathogens that were isolated in the microbiology laboratory the last 4 years.

## 2. Materials and Methods

We prospectively tested *in vitro* all isolates of *H. influenzae*, *S*. *pneumoniae*, and *M. catarrhalis* that were recovered from patients with CARTIs at the University Hospital of Heraklion over the period 1/2009–12/2012. One bacterial isolate was tested per patient. Isolates collected either from nonhospitalized patients or from hospitalized patients within 48 hours of admission were included. Organisms were recovered from patients with sinusitis, otitis media, community-acquired pneumonia, acute bacterial exacerbation of chronic bronchitis, and acute exacerbation of chronic obstructive airways disease. Blood, pleural fluid, bronchoalveolar fluid, sputum, middle ear fluid, and sinus aspirate cultures were considered acceptable sources for study isolates.

### 2.1. Identification and Antimicrobial Susceptibility Testing

*S. pneumoniae* was identified on the basis of colony and microscopic morphology, hemolytic reactions on sheep blood agar medium, catalase test, optochin susceptibility, bile solubility, and biochemical profile using the Vitek 2 automated system (BioMérieux). *H. influenzae* was identified on the basis of hemolytic reactions on horse blood agar, growth requirement for X and V factors, and by the use of Vitek 2 automated system (BioMérieux, Marcy l' Etoile, France). Identification of *M. catarrhalis* was based on colony and microscopic morphology, oxidase test, and by the use of Vitek 2 automated system (BioMérieux). For *S. pneumoniae* isolates, minimum inhibitory concentrations (MICs) were determined using the *E*-test method, for penicillin, cefuroxime, cefotaxime, ceftriaxone, cefepime, imipenem, meropenem, erythromycin, clarithromycin, roxithromycin, azithromycin, clindamycin, ciprofloxacin, levofloxacin, moxifloxacin, chloramphenicol, tetracycline, cotrimoxazole (SXT), and vancomycin. Results were interpreted according to the 2012 Clinical and Laboratory Standards Institute criteria (CLSI) [9]. The double-disk diffusion method with erythromycin (15 *μ*g) and clindamycin (2 *μ*g) (Bio-Rad, Marnes-la-Coquette, France) and MIC data were used for the determination of macrolide resistance phenotype [9]. Resistance to both erythromycin and clindamycin is characteristic of a cMLS_B_ phenotype, while blunting of the clindamycin inhibition zone near the erythromycin disc is indicative of an iMLS_B_ phenotype. Resistance to erythromycin and susceptibility to clindamycin with no blunting are characteristic of the M phenotype. Multiresistance was defined as resistance to three or more classes of antibiotics.

For *H. influenzae* and *M. catarrhalis* antimicrobial susceptibility tests were performed by employing the disk diffusion method and the results were interpreted according to the 2012 CLSI criteria [9, 10]. Intermediate isolates were grouped along with resistant isolates. The antibiotics that were tested against *H. influenzae* isolates are the following: ampicillin, amoxicillin + clavulanic acid, clarithromycin, chloramphenicol, tetracycline, SXT, ciprofloxacin, ofloxacin, and moxifloxacin. The antibiotics that were tested against *M. catarrhalis* isolates are the following: amoxicillin, amoxicillin + clavulanic acid, chloramphenicol, tetracycline, clarithromycin, SXT, rifampicin, ciprofloxacin, and moxifloxacin. Beta-lactamase (BL) production was assayed by the nitrocefin test (Oxoid, Basingstoke, UK). BLNAR *H. influenzae* strains were defined as BL-negative strains that were resistant to ampicillin (MIC ≥ 4 *μ*g/mL) [9].

In order to test for differences in the antibiotic susceptibility patterns between the earlier and later study years for a given antibiotic, we compared the resistance profiles of respiratory isolates of the period 2009-2010 with those of the period 2011-2012.

### 2.2. Statistical Analysis

Statistical analysis was conducted by Fisher's exact and Kruskal-Wallis tests, as appropriate. Statistical significance was set at *P* < 0.05. All analyses were performed with Graphpad Prism, V.4 (GraphPad Software Inc., CA, USA).

## 3. Results

### 3.1. Bacterial Isolates and Patients' Demographics

A total of 261 *S. pneumoniae*, 260 *H. influenzae*, and 67 *M. catarrhalis* were isolated during the period 1/2009–12/2012 from patients with CARTIs. The distribution of respiratory pathogens by study year is shown in Table 1. Sixty-three percent of all isolates were obtained from male patients (Table 2). Most of the *S. pneumoniae* strains were most frequently isolated from pediatric patients ≤ 5 years of age, while *H. influenzae* and *M. catarrhalis* were derived from adults ≥ 61 years of age (Table 2).

### 3.2. Antibiotic Resistance among *S. pneumoniae* Isolates

*In vitro* susceptibility data for 19 antimicrobial agents are presented in Table 3. The proportion of penicillin nonsusceptible (PNSP) isolates was 39.5% (27.2% with intermediate resistance and 12.3% with high-level resistance). Isolates with intermediate and high-level resistance became more frequent over time, from 24.3% over the period 2009 to 2010, increased to 30.4% over the years 2011 to 2012, while isolates with high-level resistance decreased the second half of the study period (*P* = 0.09) (Table 3). Resistance to the third generation cephalosporins decreased over the two two-year time periods, from 6.6% to 1.6% for cefotaxime and from 5.9% to 0.8% for ceftriaxone. Similarly, there was a trend for nonsignificant decrease in cefepime resistance (including intermediately and fully resistant strains) that decreased from 18.4% to 16% (*P* = 0.15). Resistance to erythromycin was detected in 76 isolates (29.1%). Among them, M and cMLS_B_ phenotypes were observed in 40 (52.6%) and 22 (28.9%) strains, respectively, by the use of the double disk approximation test. Additionally, 14 strains (18.4%) with the iMLS_B_ phenotype were detected. Erythromycin resistance decreased from 29.4% over the years 2009 to 2010 to 28.8% over the period 2011 to 2012, but this was not significant (*P* = 1.00) (Table 3). Only two isolates, one in each study period, were resistant to newer fluoroquinolones, while all isolates were susceptible to vancomycin (Table 3).

Multiresistance was observed among 56 (54.4%) PNSP strains. The predominant phenotype of multidrug resistance was nonsusceptible to penicillin, erythromycin, clindamycin, and tetracycline (28.6%), and the second most frequent phenotype was nonsusceptible to penicillin, erythromycin, tetracycline, and SXT (26.8%) (Table 4).

### 3.3. Antibiotic Resistance among *H. influenzae* Isolates

Of the 260 *H. influenzae *isolates, 36 (13.8%) produced *β*-lactamase. Ampicillin-resistant isolates represented 14%, while amoxicillin + clavulanic acid-resistant isolates were only 0.8%. Overall, 1 BLNAR strain was isolated. A nonsignificant increase in resistance of *H. influenzae* isolates was noted between the two study periods for ampicillin and amoxicillin + clavulanic acid. The same was true for clarithromycin, SXT, and tetracycline (Table 5).

### 3.4. Antibiotic Resistance among *M. catarrhalis* Isolates

Among the 67 isolates tested, almost half of them (32 isolates) were resistant to ampicillin and produced beta-lactamase. Macrolide resistance decreased from 36.4% in 2009-2010 to 29.4% in 2011-2012. The reverse trend was noted for cotrimoxazole. Only one isolate was resistant to tetracycline and all 67 isolates were susceptible to amoxicillin + clavulanic acid, chloramphenicol, rifampicin, and the two fluoroquinolones tested (Table 5).

## 4. Discussion

The results of the present study provide an update on resistance trends amongst pathogens that cause CARTIs in our area. Increasingly, resistance of *S. pneumoniae* to penicillin is of special concern, because this agent has been considered the therapy of choice for pneumococcal CARTIs, due to its efficacy and low cost [4, 6]. Although resistant strains of *S. pneumoniae* are detected universally, the incidence of resistance varies markedly between countries and regions. Data from the Alexander project reported penicillin resistance levels in *S. pneumoniae* ranging from 7.8% in Eastern Europe through 25% in the USA to 38.8% in several parts of Asia in 1998–2000 [4].

The first study conducted in Greece in the early 1990, evaluating the antimicrobial susceptibilities of 1,002 clinical isolates of *S. pneumoniae* deriving from patients with community-acquired pneumonia, reported resistance rates of 14% for penicillin [11]. A recent study from the same centre showed an increase of 4.5% in penicillin resistance [12]. In the present study, the percentage of intermediately resistant and resistant isolates decreased from 41.9% to 36.8% over the two study periods. More specifically, the percentage of highly resistant isolates dropped from 17.6% to 6.4%. Additionally, the low rates of resistance to third-generation cephalosporins favour their use as the first-choice empirical treatment for community-acquired pneumonia necessitating hospital admission [13]. In the present study 54.4% of PNSP strains were found to be multidrug resistant, a rate similar to that previously reported [14]. Riedel et al. in their study in 15 European countries reported overall MDR to 15.8%, varying from 0% in Denmark to 42.9% in Greece [15].

Resistance to macrolides, which reached 29.1% among pneumococcal isolates, renders empirical monotherapy with a macrolide not appropriate for treatment of patients with community-acquired pneumonia. The US (IDSA/ATS) guidelines recommend that in regions where macrolide-resistance exceeds 25%, macrolide monotherapy is not appropriate even for patients without comorbidities [16]. The M phenotype encoded by the mef gene predominated in Crete, a feature also prevailing in other areas in Greece, in the United States, and Canada but much rarer in other European countries, where the MLS_B_ phenotype, characterized by resistance to macrolides, lincosamides, and streptogramin, is most commonly encountered [17–19]. The high frequency of resistance to macrolides is due to their increased outpatient use for CARTIs. The relationship between macrolide resistance in *S. pneumoniae* and macrolide consumption has been shown by several investigators, reporting low resistance rates in low consuming areas as Scandinavia and high in Mediterranean countries with increased macrolide use, such as France, Spain, and Italy, where the respective prevalence are 58%, 29–35%, and 32%–43% [15, 20–22].

Overall, 52 isolates (19.9%) were resistant to cotrimoxazole, which is lower than that found in our previous study of the period 2001–2008 [14]. This finding is likely due to the decreased outpatient consumption of the drug in our area, since the connection between decreased consumption of SXT and decreased resistance in *S. pneumoniae* has been shown [23].

Fluoroquinolone resistance was rare among the *S. pneumoniae* isolates. In Greece, quinolone nonsusceptibility is low, although fluoroquinolones are widely used in the treatment of community-acquired infections [14, 17]. Conflicting reports exist regarding the association of fluoroquinolone use and their resistance. Some investigators reported low fluoroquinolone resistance rates despite high usage, while others found that increased usage of fluoroquinolones strictly correlates with increased incidence of resistance [24–27]. It has been also shown that the use of quinolones with limited antipneumococcal activity favours the emergence of resistant strains [28], while the more active fluoroquinolones, such as moxifloxacin and gatifloxacin, are potentially less likely to select for resistance mutations, if mutation prevention concentrations are considered in relation to achievable serum levels [29].

Regarding *H. influenzae*, production of *β*-lactamase in the current 4-year study was found in 13.8% of the isolates, which is higher than the proportion shown previously [30] but similar to that reported in the Alexander Project [31]. Within the European countries, there is considerable variability in the prevalence of *β*-lactamase production by *H. influenzae, *with previous studies showing values from <10% for Germany, Italy, The Netherlands, and Poland to >30% for France, Portugal, and Romania [31, 32]. Interestingly, it has been suggested that *β*-lactamase prevalence in *H. influenzae* has decreased in recent years in some countries [7]. Only one BLNAR strain of *H. influenzae* was detected during the study period. Although the global prevalence of BLNAR *H. influenzae* strains is very low, their incidence is on rise [33, 34].

Almost one-third of the *H. influenzae* isolates were found resistant to macrolides, a finding consistent with that of other investigators, who also used pharmacokinetic/pharmacodynamic breakpoints to interprete susceptibility data for macrolides [35, 36]. There was a noticeable decrease in the resistance of *H. influenzae *to SXT when the results of this study, where 72.7% of the isolates were susceptible to SXT, were compared with those from a previous study from the same area, where 62.3% of the isolates were susceptible to SXT [30]. This is likely due to the decreased use of SXT during the study period, although no significant connection between SXT use and resistance among *H. influenzae* isolates has been shown [23]. Tetracycline resistance was low (8%), reflecting the tendency in recent years not to use older tetracyclines for the treatment of CARTIs. All isolates are susceptible to fluoroquinolones. Fluoroquinolones still remain among the antimicrobial agents that are most powerful against *H. influenzae in vitro* and are also highly effective as treatment of respiratory tract infections [37], although resistance has been recognized [38], and therapeutic failures in patients with community-acquired pneumonia have been associated with levofloxacin resistance in *H. influenzae* [39]. Overall, 47.8% of the *M. catarrhalis* isolates were *β*-lactamase positive. The *β*-lactamases which confer resistance to ampicillin have been characterized as BRO-1 and BRO-2 for *M. catarrhalis* and are inhibited by clavulanic acid and the combination of amoxicillin + clavulanic acid has been shown to be highly active against these species [40]. Since the first report of *β*-lactamase-producing *M. catarrhalis* in 1977 [41], the percentage of these strains has increased worldwide over the years, exceeding 90% in some countries [8, 31, 32]. Apart from macrolides and cotrimoxazole all antimicrobials had good *in vitro* activity against this organism.

In conclusion, there is a decreasing trend in the prevalence of resistance of the three most common pathogens involved in CARTIs. However, continuous surveillance at local and national levels remains important to detect any further changes in pathogens and monitor any changes in antimicrobial susceptibility among the major respiratory pathogens responsible for CARTIs.

## Figures and Tables

**Table 1:** Distribution of community-acquired respiratory pathogens by study year (2009–2012).

	2009	2010	2011	2012	2009–2012
*S. pneumoniae *	73 (42.5%)	63 (49.6%)	62 (44%)	63 (42.6%)	261 (44.4%)
*H. influenzae *	79 (45.9%)	51 (40.2%)	61 (43.2%)	69 (46.6%)	260 (44.2%)
*M. catarrhalis *	20 (11.6%)	13 (10.2%)	18 (12.8%)	16 (10.8%)	67 (11.4%)

Total	172	127	141	148	588

**Table 2:** Numbers of isolates of *H. influenzae*, *S. pneumoniae*, and *M. catarrhalis*, grouped according to gender and age.

	*S. pneumoniae* (*n* = 261)	*H. influenzae* (*n* = 260)	*M. catarrhalis* (*n* = 67)
Gender			
Male	166	165	43
Female	95	95	24
Age groups (years)			
0–5	110	49	12
6–15	24	46	11
16–45	24	47	9
46–60	26	25	5
≥61	77	93	30

**Table 3:** Comparison of antibiotic resistance rates in *S. pneumonia* isolates over the periods 2009-2010 and 2011-2012.

	2009-2010							2011-2012					
Antibiotic	MIC_50_	MIC_90_	Range	*S*%	*I*%	*R*%	MIC_50_	MIC_90_	Range	*S*%	*I*%	*R*%	*P* value
Penicillin	0.023	2	<0.016–4	58.1	24.3	17.6	0.023	0.75	<0.016–4	63.2	30.4	6.4	0.09
Cefuroxime	0.023	3	<0.016–6	72.1	1.4	26.5	0.023	2	<0.016–4	80.8	2.4	16.8	0.10
Cefotaxime	0.023	1	<0.016–2	93.4	5.9	0.7	0.023	0.55	<0.016–1.5	98.4	1.6	—	0.10
Ceftriaxone	0.023	1	<0.016–1.5	94.1	5.9	—	0.023	0.5	<0.016–1	99.2	0.8	—	0.03
Cefepime	0.064	2	0.0023–3	81.6	14.7	3.7	0.125	2	0.016–6	84	7.2	8.8	0.15
Imipenem	0.016	0.19	0.003–0.75	88.2	10.3	1.4	0.047	0.125	0.006–0.5	94.4	5.6	—	0.10
Meropenem	0.012	0.38	0.004–1	88.2	8.8	2.9	0.032	0.125	0.006–1	96	2.4	1.6	0.14
Erythromycin	0.094	≥256	0.047–≥256	70.6	—	29.4	0.064	≥256	0.016–≥256	71.2	—	28.8	1.00
Clarithromycin	0.094	≥256	0.016–≥256	70.6	—	29.4	0.047	≥256	0.016–≥256	71.2	—	28.8	1.00
Clindamycin	0.125	≥256	0.032–≥256	89.7	—	10.3	0.064	≥256	0.023–≥256	82.4	1.6	16	0.10
Roxithromycin	0.125	≥256	0.047–≥256	70.6	—	29.4	0.094	≥256	0.016–≥256	71.2	—	28.8	1.00
Azithromycin	0.5	≥256	0.125–≥256	70.6	—	29.4	0.19	≥256	0.023–≥256	71.2	—	28.8	1.00
Ciprofloxacin	0.75	1.5	0.385–≥32	99.3	—	0.7	0.75	1	0.25–≥32	99.2	—	0.8	1.00
Levofloxacin	0.75	1	0.19–16	99.3	—	0.7	0.75	1	0.19–≥32	99.2	—	0.8	1.00
Moxifloxacin	0.125	0.19	0.032–1.5	99.3	0.7%	—	0.125	0.19	0.047–3	99.2	—	0.8	0.16
Chloramphenicol	2	2	0.38–12	99.3	—	0.7	2	3	0.75–32	96.8	—	3.2	0.19
Tetracycline	0.125	16	0.016–48	74.3	1.4	24.3	0.25	32	0.064–64	79.2	—	20.8	0.10
Cotrimoxazole	0.19	1.5	0.016–≥32	77.2	14.7	8.1	0.094	1.5	0.023–≥32	83.2	9.6	7.2	0.10
Vancomycin	0.38	0.5	0.25–0.5	100	—	—	0.38	0.5	0.25–0.75	100	—	—	NA*

*NA: not applicable.

**Table 4:** MDR phenotypes of the 56 pneumococcal isolates by study year.

MDR phenotype	2009	2010	2011	2012
P, E, Te	2	2	1	2
P, E, SXT	1	1	0	0
P, E, CM, Te	1	4	7	4
P, E, CM, SXT	0	0	0	1
P, E, Te, SXT	6	5	4	0
P, E, CM, Te, SXT	1	4	3	2
P, E, CM, C, Te, SXT	0	1	3	0
P, C, Te, SXT	0	0	0	1

Total	11	17	18	10

P: penicillin; E: erythromycin; Te: tetracycline; SXT: cotrimoxazole; CM: clindamycin; C: chloramphenicol.

**Table:** (a) *Haemophilus influenza *

Antimicrobial agent	2009 *n* = 79 resistant	2010 *n* = 51 resistant	2011 *n* = 61 resistant	2012 *n* = 69 resistant	2009-10 (A) *n* = 130 resistant	2011-12 (B) *n* = 130 resistant	*P* value A versus B
Ampicillin	11 (13.9%)	5 (9.8%)	10 (16.4%)	10 (14.5%)	16 (12.3%)	20 (15.4%)	0.59
Amoxicillin + CA	0 (0%)	1 (2%)	0 (0%)	1 (0.7%)	1 (1.4%)	1 (1.4%)	1.00
Clarithromycin	25 (31.6%)	15 (29.4%)	17 (27.9%)	21 (30.4%)	40 (30.8%)	38 (29.2%)	0.89
Chloramphenicol	0 (0%)	0 (0%)	0 (0%)	0 (0%)	0 (0%)	0 (0%)	NA
Tetracycline	6 (7.6%)	4 (7.8%)	5 (8.2%)	6 (8.7%)	10 (7.7%)	11 (8.5%)	1.00
Cotrimoxazole	18 (22.8%)	15 (29.4%)	19 (31.1%)	19 (27.5%)	33 (25.4%)	38 (29.2%)	0.57
Ciprofloxacin	0 (0%)	0 (0%)	0 (0%)	0 (0%)	0 (0%)	0 (0%)	NA
Ofloxacin	0 (0%)	0 (0%)	0 (0%)	0 (0%)	0 (0%)	0 (0%)	NA
Moxifloxacin	0 (0%)	0 (0%)	0 (0%)	0 (0%)	0 (0%)	0 (0%)	NA

CA: clavulanic acid; NA: not applicable.

**Table:** (b) *Moraxella catarrhalis *

Antimicrobial agent	2009 *n* = 20 resistant	2010 *n* = 13 resistant	2011 *n* = 18 resistant	2012 *n* = 16 resistant	2009-10 (A) *n* = 33 resistant	2011-12 (B) *n* = 34 resistant	*P* value A versus B
Am*ο*xicillin	8 (40%)	8 (61.5%)	8 (44.4%)	8 (50%)	16 (48.5%)	16 (47%)	1.00
Amoxicillin + CA	0 (0%)	0 (0%)	0 (0%)	3 (18.7%)	0 (0%)	0 (0%)	NA
Chloramphenicol	0 (0%)	0 (0%)	0 (0%)	0 (0%)	0 (0%)	0 (0%)	NA
Tetracycline	0 (0%)	0 (0%)	0 (0%)	1 (6.3%)	0 (%)	1 (2.9%)	1.00
Clarithromycin	7 (35%)	5 (38.5%)	4 (22.2%)	6 (37.5%)	12 (36.4%)	10 (29.4%)	0.60
Cotrimoxazole	6 (30%)	2 (15.4%)	5 (27.8%)	6 (37.5%)	8 (24.2%)	11 (32.3%)	0.59
Rifampicin	0 (0%)	0 (0%)	0 (0%)	0 (0%)	0 (0%)	0 (0%)	NA
Ciprofloxacin	0 (0%)	0 (0%)	0 (0%)	0 (0%)	0 (0%)	0 (0%)	NA
Moxifloxacin	0 (0%)	0 (0%)	0 (0%)	0 (0%)	0 (0%)	0 (0%)	NA

CA: clavulanic acid; NA: not applicable.
